# Cook County Hospital Surgical Clinics

**Published:** 1866-08

**Authors:** R. G. Bogue

**Affiliations:** Attending Surgeon to the Hospital


### HOSPITAL REPORTS.

*Cook County Hospital Surgical Clinics.* By R.G. Bogue, M.D., Attending Surgeon to the Hospital.

### CASE I. FROST-BITE OF THE EXTREMITIES—AMPUTATION OF THE HAND AND THE FOOT—RECOVERY.

February 10th, 1866.

Gentlemen,—The case we show you to-day is one of gangrene of the foot and hand resulting from frost-bite. The House Surgeon, Dr. Quales, will read his notes of the case:

Edward McGeary, aged 26, late from the army, where he lost his right leg, was admitted January 20th, 1866. On the morning of admission, he was found in an alley, insensible, stiffened from cold, having been exposed probably during the greater part of the night.

When admitted, he was insensible. His jaws were firmly set. Right thigh off, about four inches above the knee. Left foot and both hands rigidly frozen. Respiration 12 per minute. Pulse imperceptible. Heart's action very feeble.

*Treatment.*—The frozen extremities were placed in cold water. Friction was applied steadily. Brandy was administered internally, with some difficulty, however, on account of his teeth being firmly clenched. As soon as the frozen limbs were thawed, he was removed to a warm room. Dry friction was applied continually to the whole body; stimulants were administered internally. After a lapse of about three hours, during which time friction and warmth were constantly applied, he evinced signs of returning consciousness. He finally recovered so as to articulate coherently. The frozen extremities were now enveloped in cotton, and were covered with oiled silk. Milk-punch and beef-tea.

*Progress of the Case.*—Jan. 21. Very restless during the night; suffers intense pain; pulse 100 per minute; feeble respiration, 20 per minute; stomach very irritable; ejected even small quantities of cold water. Ordered to take morph. sulph.

gr. $\frac{1}{4}$ every four hours. Milk-punch, ℥i. every two hours, also beef-tea.

Jan. 22. Vomiting ceased. Rested pretty well during the night. No appetite; complains of great pain. Ordered quin. sulph. gr. i., pulv. opii, gr. $\frac{1}{2}$, every four hours. Continue milk-punch and beef-tea.

Jan. 26. Frozen parts commenced sloughing. A poultice was substituted for the previous dressing. Ordered to take quin. sulph. grs. ii. and tinct. ferri chlorid. gtt. x. thrice daily. Bowels not having moved for two days, he was ordered to take ol. ricini, ℥ss. at bedtime.

Feb. 3. Line of demarkation very distinct. On the left hand, the sloughing involves all the fingers extending on the palmar surface as high as a line corresponding with the junction of the lower and middle third of the metatarsal bones; posteriorly, it involves the whole of the dorsum as far as the carpal articulation. On the right hand there is no sloughing, only superficial abrasions over the finger-joints and around the nails. There is considerable numbness, however, and very limited flexor and extensory power. On the foot, the sloughing involves the toes and the tissues as far as the lower half of the metatarsal bones.

The effect of excessive cold applied to a part, is to impair or destroy its vitality. Where the degee of cold is not too great, the vitality is impaired; the part is debilitated; a state of chronic congestion is established, so that the limb is affected by even a moderate degree of cold; leaving it in the condition of pernio or chilblain. In cases where the cold has been more severe, superficial sloughing is the result, producing ulcers which are slow to heal. Again, the cold may be so severe, and may be applied for so long a time, that all the tissues are destroyed, and extensive sloughing follows. The amount of sloughing often depends upon the mode of treatment adopted. If reaction is very gradual, many of the evil consequences may be avoided; but if it is allowed to progress rapidly, inflammation will be excessive, and the sloughing will be extensive, involving, as in this case, nearly the whole hand or foot, or even an entire limb.

The parts most liable to frost-bite are the parts most distant from the centre of circulation, viz., the toes and feet, fingers and hands, the ears and the nose.

At first, while the part is frozen and stiff, snow, or water made cold by the addition of snow or ice, should be applied to retard reaction. When reaction is established, simple, cool anodyne dressings should be used—cool water, with tr. opii, ℥j. to the pint. After a few days, if the danger of sloughing is past, moderately stimulating applications may be employed; *e. g.*, tr. iodini, ʒj., aquæ, ℥iv.—on an aqueous solution of iodine and iodide of potassium—grs. ij. or iij. of the former and grs. x. of the latter to the ℥ of water. Either of these may be painted on the part two or three times a day until it has recovered. When the effect of the cold is more profound, and there is to be sloughing, it is better to apply a poultice to favor the recuperation of the dead tissues. These applications may be rendered antiseptic by the addition of yeast or pulverized charcoal or the liquor sodæ chlorinatæ. Their use must be continued until a line of demarkation separates the gangrenous portion from the healthy. The proper time for amputation has then arrived.

This patient has already lost one limb above the knee, from a gunshot injury, therefore it is desirable to save for him a part of the remaining foot if possible. If the state of the tissues allowed, we would leave him the stump of Chopart's amputation. Syme's amputation through the ankle-joint is inadmissable, on account of the slough upon the heel. We must go above the ankle. The condition of the hand is such, that it will be necessary to remove the whole member.

[After the administration of ether, the hand was removed by a cut through the lower third of the forearm, making anterior and posterior flaps. On examination of the foot, pus was found burrowing along the sheaths of the tendons and under the plantar fascia to such an extent, that a proper flap could not be made for Chopart's operation. Lenoir's operation was performed above the ankle, making anterior and posterior flaps, and removing the malleoli, with the articulating surface of the tibia, about the same as in Syme's operation at the ankle-joint. The arteries in both stumps were ligated; the flaps were adjusted with interrupted sutures, and cold water dressings applied. Reaction occurred favorably, and the patient made a perfect recovery, the stumps healing by granulation, and being cicatrized, March 20th.]

CASE II. FROST-BITE OF BOTH FEET—DOUBLE AMPUTATION—OSTEO-MYELITIS—DEATH.

March 23d, 1866.

After the House Surgeon has related the history of the footless cadaver before you, we will examine the condition of the stumps of the limbs upon which you saw me operate three weeks ago, and I will contrast the case with the subject of our previous operation.

John Galvia, aged 28, admitted February 21st, 1866, states that about five years ago he received a gunshot wound in the right hip, causing him considerable injury; but, after suffering for eight months, he recovered with but little shortening of the limb. Two years subsequently he was run over by a wagon, injuring the same limb and producing compound fracture of its femur; but, with careful treatment, at the expiration of ten months, he again recovered with the loss of several pieces of the bone and with eight inches shortening. He also states that four days previous to admission, while coming from the country to the city, he froze his feet.

*Symptoms on Admission.*—Physical powers somewhat depressed by recent dissipation and exposure. Right leg eight and a half inches shorter than the left. Both feet recently frozen; inflamed and swollen. Pulse, 95 per minute. Appetite good.

*Treatment.*—Cold water dressings to the feet, with an anodyne at night.

*Progress of the Case.*—February 25th. Inflammation somewhat subsided. Ordered warm poultices to the feet, and quin. sulph. ℈i. ss., tinct. ferri mur. ʒii., aquæ ℥ii. M. S. Give ʒii. three times per diem.

March 1st, 1866. Line of demarkation fully defined: on the left foot, at the junction of the lower with the middle third of the metatarsal bones, and on the right, extending on the dorsum of the foot to the tarso-metatarsal joints, including also the heel and plantar surface.

*Operation.*—March 2d. Dr. R. G. Bogue performed Chopart's operation on the left foot, but on the right limb was compelled to amputate through the tibia and fibula at their lower third.

*Progress of the Case.*—March 3d. Continue tonics, quiniæ sulph. and tinct. ferri chlor. Anodynes were administered last night, but failed to induce sleep. Flaps looking well. Ate breakfast with relish.

March 8th. Flaps healed partially by immediate union, with comparatively little suppuration or inflammation, but complains of intense pain to-day in the right leg.

March 12th. Had a rigor during the night. Stumps have nearly ceased suppurating. Pulse weak and accelerated; 105 per minute. Brandy, ℥i. every four hours.

March 15th. Seeming better. Ordered to continue tonics, stimulants and extra diet. Bowels being constipated, gave the following: Extract colocynth. comp., ext. taraxaci, aa. ℈i., ext. hyosciam. grs. x. Misce. F. pil. No. x. S. Give one night and morning.

March 18th. Patient presents every symptom of pyæmia, with occasional delirium. ℞. Sodæ sulphis. grs. v. every four hours, in solution; also, quin. sulph. grs. x., ext. hyosciam. grs. v. M. F. pil. No. x. Give one every four hours, alternated with the soda; also, milk-punch, ℥viii. thrice daily. Continue beef-tea; omit quiniæ and iron.

March 19th. ℞. Quiniæ sulph., ammon. carbon. aa. grs. v. every four hours. Cont. soda and stimulants.

March 21st. Comatose. Involuntary evacuations. The discharge from the stumps is dark-colored and ichorous.

March 22d. Continued perfectly comatose, and succumbed at 6 o'clock P. M.

The body is greatly emaciated, and of a sallow hue. On opening the thorax, the heart is found normal. On the anterior surface of the inferior lobe of the right lung, there are signs of recent inflammation and exudation. There are no abnormal appearances in the stomach or intestines. Both kidneys and the liver exhibit well-marked fatty degeneration.

On laying open the flaps of the right stump, you see incipient caries of the tibia and the fibula; also that protrusion of the medullary membrane and medulla, which is caused by osteomyelitis. In the left stump there is erosion of the anterior surfaces of the os calcis and astragalus; also numerous abscesses and burrowing sinuses within the flap. The margin of the right acetabulum is almost wholly absorbed. The cotyloid cavity is very shallow, and the head of the femur is partially distorted. Pus exists within the capsular ligament, but is probably not of recent deposit.

The condition of the stumps was more favorable in this case than in the case of McGeary. They united by adhesion through the greater portion of their extent. There was but little swelling or soreness until the fifth day, when the patient experienced excessive pain and soreness, with some swelling of the stump of the leg, extending to the knee. This subsided in the course of three or four days, under the use of warm fomentations.

In the first case, about one-third of the posterior flap sloughed, and there was some inflammation of the flaps of the forearm; yet these eventually did well, the patient making a good recovery; while, in the present instance, there was pyæmia with all its fatal consequences.

The occurrence of osteo-myelitis in the second case, may account for the development of the pyæmia. It is certain that its appearance in any case is a very unfavorable omen. Whether this precedes the blood-poisoning or follows it, is not certain:—it occurs early, in some cases, after operation; certainly, before any signs of blood-poisoning show themselves. In this case, it probably commenced when inflammation appeared in the stump. This condition of the ends of the bones and the medullary canal could not be determined at that time—not until a few days before the death of the patient, when the flaps separated at the internal commissure, revealing the necrosed end of the tibia, and the border of the myelitic mass protruding from the medullary cavity of the bone. Before that time, the flaps were united along their whole border.

If this disease is peculiarly favorable to the development of pyæmia, it would be justifiable to modify our operations so as to avoid if possible its occurrence. This may be done by amputating through the joints, or through the ends of the long bones, avoiding the medullary canal. I believe this may be done, in the leg particularly, leaving just as serviceable a stump as by cutting through the shaft, if we perform Lenoir's amputation at the ankle; or, where this cannot be done, by sacrificing the limb near enough to the knee, to avoid the canal of the tibia. In the thigh, wherever it is possible, by cutting through the condyles of the femur, or sufficiently near to avoid its canal.

I believe the frequent occurrence of this disease will eventually influence surgeons in their selection of the point of operation, and will lead them to avoid as far as possible all amputations involving the medullary canal of any of the long bones.

